# Endangered Steppe Eagle (*Aquila nipalensis*) (Aves, Accipitriformes, Accipitridae) genome and mitogenome assembly: A resource for molecular evolution and comparative genomics

**DOI:** 10.3897/zookeys.1281.158566

**Published:** 2026-06-03

**Authors:** Wannapol Buthasane, Thidathip Wongsurawat, Piroon Jenjaroenpun, Sithichoke Tangphatsornruang, Wirulda Pootakham, Chutima Sonthirod, Wasitthee Kongkachana, Alisa Wilantho, Ratiwan Sitdhibutr, Chaiyan Kasorndorkbua, Gunnaporn Suriyaphol

**Affiliations:** 1 Biochemistry Unit, Department of Physiology, Faculty of Veterinary Science, Chulalongkorn University, Bangkok 10330, Thailand Division of Medical Bioinformatics, Research Department, Faculty of Medicine Siriraj Hospital, Mahidol University Bangkok Thailand; 2 Division of Medical Bioinformatics, Research Department, Faculty of Medicine Siriraj Hospital, Mahidol University, Bangkok 10700, Thailand Biochemistry Unit, Department of Physiology, Faculty of Veterinary Science, Chulalongkorn University Bangkok Thailand; 3 National Center for Genetic Engineering and Biotechnology (BIOTEC), National Science and Technology Development Agency, Pathum Thani 12120, Thailand National Center for Genetic Engineering and Biotechnology (BIOTEC), National Science and Technology Development Agency Pathum Thani Thailand; 4 Raptor Rehabilitation Unit, Kasetsart University Veterinary Teaching Hospital Kamphaeng Saen Campus, Nakhon Pathom 73140, Thailand Raptor Rehabilitation Unit, Kasetsart University Veterinary Teaching Hospital Kamphaeng Saen Campus Nakhon Pathom Thailand; 5 Laboratory of Raptor Research and Conservation Medicine, Department of Pathology, Faculty of Veterinary Medicine, Kasetsart University, Bangkok, 10900, Thailand Department of Pathology, Faculty of Veterinary Medicine, Kasetsart University Bangkok Thailand

**Keywords:** Comparative analysis, *de novo* genome assembly, evolution

## Abstract

The Steppe Eagle, *Aquila nipalensis* Hodgson, 1833, is a migratory, endangered raptor experiencing population declines due to habitat loss and human persecution. This study aims to construct a high-quality *de novo* nuclear genome and complete mitochondrial genome assembly using PromethION Oxford Nanopore long-read and MGI short-read sequencing technologies. The assembled genome size was 1.21 Gb and contained 16,192 predicted protein-coding genes. Phylogenomic reconstruction based on ultraconserved elements (UCEs) robustly placed *A. nipalensis* within Aquilinae, with *Aquila chrysaetos* (Linnaeus, 1758) identified as its closest extant relative. Mitogenome-based analyses recovered congruent topology but revealed dataset-dependent differences in divergence time estimation. The most recent common ancestor of *A. nipalensis* and other *Aquila* species was estimated at approximately 3–13 million years ago, depending on dataset, whereas divergence between *Aquila* and *Nisaetus* occurred around 22 Mya. Comparative genomic analyses further identified positively selected genes associated with vesicle trafficking, secretion, and tissue development, suggesting potential adaptive signatures related to physiological performance. In conclusion, these genomic and evolutionary insights establish a foundational reference for future population genomic, adaptive, and conservation studies of this endangered raptor.

## Introduction

The Steppe Eagle (*Aquila nipalensis* Hodgson, 1833) is a large-bodied eagle belonging to the family Accipitridae. *Aquila nipalensis* is a long-distance migratory raptor widely distributed across the Palaearctic steppe. It breeds primarily in open grasslands of Eurasia, from southern Russia and Kazakhstan eastward through Mongolia to northern China, and winters across Africa, the Middle East, and parts of South and Southeast Asia. This extensive migratory system links breeding and wintering habitats across thousands of kilometres and exposes the species to diverse ecological pressures throughout its range (Bari et al. 2020). The species has been recorded only infrequently in Thailand during the winter season. This species is classified as Endangered in the International Union for Conservation of Nature (IUCN) Red List (BirdLife International 2020). Population declines have been attributed to the conversion of steppe habitats into agricultural land, direct persecution, and electrocution from electric lines (BirdLife International 2020). The Steppe Eagle, a diurnal raptor species and primarily preys on small mammals, birds, and reptiles, but it also scavenges on carrion (Orihuela-Torres et al. 2021). Its scavenging behaviour increases its risk of exposure to chemical contaminants, similar to vultures, with cases of diclofenac toxicity reported as contributing to population declines (Sharma et al. 2014). However, the genetic aspects related to those functions have not yet been reported in the Steppe Eagle.

Genomic research provides extensive biological information, encompassing genes associated with molecular functions, biological processes, and cellular components. It also offers valuable insights into evolutionary relationships among species (Zhang et al. 2014b; Stiller et al. 2024). Within the family Accipitridae, reference genomes have been assembled for several species such as Golden Eagle (*Aquila chrysaetos* (Linnaeus, 1758)), Bald Eagle (*Haliaeetus leucocephalus* (Linnaeus, 1766)), a White-tailed Eagle (*Haliaeetus albicilla* (Linnaeus, 1758)) and Asian King Vulture (*Sarcogyps calvus* (Scopoli, 1786)) (Zhang et al. 2014a; Mead et al. 2021; Buthasane et al. 2024). *Aquila nipalensis* belongs to the booted eagles (subfamily Aquilinae), a lineage of Accipitridae characterised by feathered tarsi and complex evolutionary relationships revealed by molecular phylogenetic studies. Early multigene studies demonstrated that traditional eagle genera such as *Aquila* Brisson, 1760, *Hieraaetus* Kaup, 1844, and *Spizaetus* Vieillot, 1816 are not strictly monophyletic, indicating that morphological classifications had obscured true evolutionary relationships. Subsequent molecular phylogenies confirmed the monophyly of the booted eagle clade while revealing deep subdivisions and taxonomic restructuring within Aquilinae. More recent comprehensive analyses based on multiple genetic loci support several major clades within the group and refined the taxonomy of booted eagles, clarifying relationships among species in *Aquila* and related genera (Helbig et al. 2005; Lerner and Mindell 2005; Lerner et al. 2017).

However, a comprehensive genomic analysis of Steppe Eagle and its comparative genomic context have not yet been reported. In addition to nuclear genomes, mitochondrial genomes (mitogenomes) are widely studied in wildlife conservation and species identification. Moreover, mitogenomes are extensively used to estimate divergence times, offering crucial insights into the molecular evolution of various avian species, such as Golden Eagle, Eurasian Goshawk (*Astur gentilis* (Linnaeus, 1758)), Red Junglefowl (*Gallus gallus* (Linnaeus, 1758)), Osprey (*Pandion haliaetus* (Linnaeus, 1758)), and Secretary Bird (*Sagittarius serpentarius* (Miller, 1779)) (Arcones et al. 2021). Understanding divergence time estimation is fundamental to unravelling the historical relationships among species, reconstructing evolutionary lineages, and assessing the accumulation of genetic variation over time (Nabholz et al. 2009; Eo and DeWoody 2010). The objective of the present study was to assemble a reference genome and mitogenome of Steppe Eagle by integrating long-read and short-read sequencing technologies. Additionally, we investigated the species’ unique genetic features, its evolutionary relationships with related species, and estimated divergence times. The findings provide essential baseline data to support future population genomic studies, including breeder selection and efforts toward population restoration.

## Materials and methods

### Genome assembly, gene prediction, and annotation

Sample collection was conducted in accordance with ethical guidelines approved by the Institutional Animal Care and Use Committee of Kasetsart University, Thailand (approval no. ACKU64-VET-052). Genomic DNA was extracted from a whole-blood sample preserved in 70% ethanol, obtained from a male Steppe Eagle housed at the Raptor Rehabilitation Unit, Kasetsart University. High molecular weight (HMW) DNA was isolated using the Wizard HMW DNA Extraction Kit (Promega, Madison, WI, USA). DNA concentration was quantified using a NanoDrop One Microvolume UV-Vis Spectrophotometer (Thermo Fisher Scientific, Waltham, MA, USA), and DNA quality and integrity were assessed via pulsed-field gel electrophoresis (PFGE). Sequencing was conducted using both long-read and short-read platforms to obtain comprehensive genomic data. ONT long-read sequencing was performed on a PromethION 2 Integrated (P2i) platform (Oxford Nanopore Technologies, Oxford, UK) using two PromethION flow cells (R10.4.1). HMW genomic DNA was used for library preparation with the ONT Ligation Sequencing Kit (SQK-LSK114; Oxford Nanopore Technologies) following the manufacturer’s protocol. Basecalling was performed using Guppy (Oxford Nanopore Technologies), and the resulting reads were exported in FASTQ format for downstream analyses. Short-read paired-end sequencing was carried out on the MGISEQ-2000 platform (MGI Tech, Shenzhen, China). Raw ONT reads were *de novo* assembled using Flye v. 2.9.1-b1780 with the ‘--meta’ option (Kolmogorov et al. 2019). The draft assembly was polished in two stages: (1) long-read polishing was performed using Racon v. 1.5.0 for two rounds with parameters -m 8 -x -6 -g -8 -w 500) (https://github.com/isovic/racon), followed by Medaka v. 1.6.1 using the r941_min_sup_g507 model (https://github.com/nanoporetech/medaka); and (2) short-read polishing was conducted with Pilon v. 1.24 (Walker et al. 2014), iterating until no further improvements were detected. Genome completeness was evaluated using BUSCO v. 4.0.5 with the avian single-copy ortholog gene set (*n* = 8,338) (Manni et al. 2021).

For homology-based gene prediction, protein sequences from four avian species—*G. gallus*, *Meleagris gallopavo* Linnaeus, 1758, *Taeniopygia guttata* Vieillot, 1817, and *Columba livia* Gmelin, 1789—retrieved from the UniProtKB database (https://www.uniprot.org) were aligned to the assembled genome using Exonerate within the MAKER annotation pipeline (Holt and Yandell 2011). The coding sequences of *A. chrysaetos chrysaetos* (Accession GCA_900496995.4) were applied as expressed sequence tag (EST) evidence in the MAKER pipeline. *Ab initio* predictions were generated using AUGUSTUS v. 3.3.3 (Augustus: Gene Prediction, RRID:SCR_008417), with parameters trained on *G. gallus* genes. Additionally, SNAP was employed for another round of *ab initio* prediction, with training performed using gene models derived from the initial round of MAKER annotation (Korf 2004). Gene filtering criteria included a minimum coding sequence length of 300 bp and a maximum intron length of 10 kb. Gene models generated from SNAP and AUGUSTUS predictions were retained only if they exhibited an Annotation Edit Distance (AED) score ≤ 0.25.

### Phylogenetic analysis using ultraconserved elements (UCEs)

Ultraconserved elements (UCEs) were extracted using PHYLUCE v. 1.6.8. The 5K UCE probe set targeting approximately 5,060 loci was downloaded from the Faircloth Lab UCE probe repository. To assess the impact of missing data, complete data matrices were generated and filtered at 75%, 95%, and 100% occupancy thresholds. Following sequence alignment and trimming, the best-fit substitution model for each filtered dataset was determined using ModelTest-NG v. 0.1.7 under the best-fit substitution models. Nodal support was assessed using 1,000 nonparametric bootstrap replicates. Phylogenetic relationships were inferred among 26 species representing the orders Accipitriformes and Cathartiformes. Details of the species included in the analysis, along with their accession numbers, are provided in Table 1. *Gallus gallus* was designated as the outgroup.

**Table 1.** Species included in the phylogenetic analyses with their GenBank accession numbers.

Scientific name	GenBank accession number	
	Genome assembly	Mitogenome
*Accipiter nisus*	GCA_027497715.1	—
*Tachyspiza soloensis*	GCA_025727755.1	—
*Tachyspiza virgata*	GCA_025728005.1	—
*Aegypius monachus*	GCA_025309335.1	—
*Aquila chrysaetos*	GCA_900496995.4	NC_024087.1
*Aquila fasciata*	—	NC_029188.1
*Aquila nipalensis*	Present study	Present study NC_045042.1
*Astur gentilis*	GCA_929443795.1	NC_011818.1
*Butastur indicus*	GCA_026770005.1	—
*Butastur liventer*	GCA_025447895.1	—
*Buteo buteo*	GCA_025504755.1	—
*Buteo hemilasius*	GCA_025727635.1	—
*Buteo lagopus*	GCA_025727815.1	—
*Cathartes aura*	GCA_000699945.1	—
*Circaetus pectoralis*	GCA_013399555.1	NC_052805.1
*Gallus gallus*	GCA_016699485.1	MG605671
*Gymnogyps californianus*	GCA_018139145.2	BK059163.1
*Gyps fulvus*	GCA_025727715.1	—
*Gyps himalayensis*	GCA_021398385.1	—
*Haliaeetus albicilla*	GCA_000691405.1	NC_040858.1
*Haliastur indus*	GCA_027575065.1	—
*Milvus migrans*	GCA_026770275.1	—
*Nisaetus alboniger*	GCA_026109245.1	NC_007599.1
*Nisaetus nipalensis*	GCA_012487455.1	NC_007598.1
*Pandion haliaetus*	GCA_013401275.1	NC_008550.1
*Sagittarius serpentarius*	GCA_013399415.1	NC_023788.1
*Sarcogyps calvus*	GCA_040869245.1	OR896160.1
*Spizaetus tyrannus*	GCA_013399215.1	NC_052803.1

### Mitogenome assemblies, annotations, and phylogeny inference

The mitogenome was sequenced using the MGISEQ-2000 platform (MGI Tech, Shenzhen, China) and assembled with NOVOPlasty v. 3.8.2 (Dierckxsens et al. 2017). Annotation was carried out using the MITOS WebServer (Bernt et al. 2013). Protein-coding, rRNA and tRNA genes were further identified using the NCBI BLAST (Altschul et al. 1990). The circular structure of the mitogenome was visualized using OrganellarGenomeDRAW (OGDRAW) v. 1.3.122 (Greiner et al. 2019). Analyses of nucleotide and amino acid composition were conducted using MEGA X (Kumar et al. 2018).

Two mitochondrial DNA datasets were generated for phylogenetic tree analyses. In the first dataset, the complete mitogenome of *A. nipalensis* was compared with 13 previously published mitogenomes representing diverse avian orders, including Accipitriformes, Cathartiformes and Galliformes. Maximum-likelihood (ML) phylogenies were reconstructed using the concatenated nucleotide sequences of the 13 conserved mitochondrial protein-coding genes (PCGs): cytochrome b (*CYTB*); NADH dehydrogenase subunits 1 (*ND1*), *ND2*, *ND3*, *ND4*, *ND4L*, *ND5*, and *ND6*; cytochrome c oxidase subunits 1 (*COX1*), *COX2*, and *COX3*; and ATP synthase F0 subunit 6 (*ATP6*) and *ATP8*. The newly sequenced mitogenome of *A. nipalensis* were included in both phylogenetic reconstruction and divergence time estimation. Details of the species included in in the analyses, along with their GenBank accession numbers, are provided in Table 1.

Multiple sequence alignments were performed using MAFFT v. 7.505, and poorly aligned regions were trimmed with trimAl v. 1.5.rev1. The best-fit nucleotide substitution models were selected using ModelFinder: GTR+F+I+G4 for the concatenated PCG dataset. ML phylogenetic trees were inferred using IQ-TREE v. 2.4.0 with 1,000 ultrafast bootstrap replicates (Nguyen et al. 2014; Hoang et al. 2017). The resulting trees were visualised in FigTree v. 1.4.4 (Rambaut 2018), with Galliformes species used as the outgroup.

Divergence times were estimated using MCMCTree implemented in the PAML v. 4.9j package. The gradient and Hessian matrix were calculated using CODEML, and analyses were performed with a burn-in of 1,000 iterations. A secondary fossil calibration point was constructed using divergence constraints between *A. chrysaetos* and *G. gallus*, *Sagittarius serpentarius* and *G. californianus*, *A. chrysaetos* and *P. haliaetus*, and *A. chrysaetos* and *Astur gentilis*. Minimum and maximum bounds of 86.5-101.8, 56.9-90.0, 37.7-59.0, and 8.1-50.1 Mya, respectively, were obtained from the TimeTree database (Kumar et al. 2022). Taxonomic nomenclature followed Gregory et al. (2024).

### Positively selected gene (PSG) analysis

To identify genes under positive selection, coding sequences have been analysed from six accipitriform species, including *A. nipalensis*, *A. chrysaetos*, *Spizaetus tyrannus*, *Astur gentilis*, *H. albicilla*, and *Sarcogyps calvus*, with *G. gallus* (Galliformes) serving as the outgroup. Positive selection analyses were conducted using the branch-site model in CODEML, implemented via PosiGene (Sahm et al. 2017). Candidate PSGs were identified based on a non-synonymous to synonymous substitution ratio (dN/dS > 1) and a significance threshold of False Discovery Rate (FDR) correction (*Q*-value) < 0.05. Functional annotation and enrichment analyses of significant PSGs were conducted using the DAVID database to classify associated Gene Ontology (GO) biological processes, molecular functions, and cellular components (Huang et al. 2009; Sherman et al. 2022).

## Results

### *De novo* genome assembly and annotation

The Steppe Eagle reference genome was assembled using raw reads generated from ONT sequencing (36× coverage; 43.90 Gb total bases) and the MGISEQ-2000 platform (117× coverage; 142.05 Gb total bases). The final assembled genome was 1.21 Gb in length (154 × total coverage) and has been deposited in GenBank under accession number PRJNA916808. After removing low-quality and duplicated contigs, the assembly achieved a scaffold N50 of 23.49 Mb. Genome completeness assessed using BUSCO v. 4.0.5 with the Aves dataset (8,338 single-copy orthologs) revealed 97.2% complete BUSCOs, including 96.9% single-copy and 0.3% duplicated BUSCOs, along with 0.6% fragmented and 2.2% missing BUSCOs. Detailed genome assembly statistics and repeat element composition are summarized in Tables 2, 3. Genome annotation identified 16,192 protein-coding genes with an average gene length of 14,196 bp. On average, each gene contained 10.02 exons and 7.79 introns. Detailed gene structure statistics, including exon and intron lengths and GC content, are summarized in Table 4.

**Table 2.** Genome assembly statistics for *Aquila nipalensis*.

Statistic	Value
N50 (bp)	23,485,944
L50	18
N70 (bp)	13,799,119
L70	32
N90 (bp)	6,409,290
L90	57
Total assembly size (bp)	1,206,752,884
Number of contigs/scaffolds	1,036
Contigs/scaffolds ≥ 100 kbp	147
Contigs/scaffolds ≥ 1 Mbp	86
Contigs/scaffolds ≥ 10 Mbp	44
Longest contig/scaffold (bp)	52,297,487
Percentage of ambiguous bases (%N)	0
GC (%)	42.29
BUSCO completeness (%) (Aves_odb10)	97.2
BUSCO duplicated (%)	0.3
BUSCO fragmented (%)	0.6
BUSCO missing (%)	2.2

**Table 3.** Summary of repetitive elements in the genome of *Aquila nipalensis*.

Repeat element	Number of elements	Total length (bp)	Percentage of genome (%)
Retrotransposons			
LINE	83,855	32,712,501	2.71
SINE	2	151	0.00
LTR	14,512	4,268,267	0.35
DNA transposons	4,706	346,655	0.03
Simple repeats	279,202	10,560,709	0.88
Low complexity	51,803	2,554,678	0.21
Unclassified	124,764	42,392,404	3.51

**Table 4.** Summary statistics of gene annotation in the genome of *Aquila nipalensis*.

Feature	Value
Protein-coding genes	16,192
Total gene length (bp)	228,000,000
Average gene length (bp)	14,196
Total exon length (bp)	28,230,000
Average exon length (bp)	178
Total intron length (bp)	201,040,000
Average intron length (bp)	1,377
Average exons per gene	10.02
Average introns per gene	7.79
GC content in exons (%)	51.73
GC content in introns (%)	42.22

### UCE-based phylogenomic analysis

**Figure 1.**
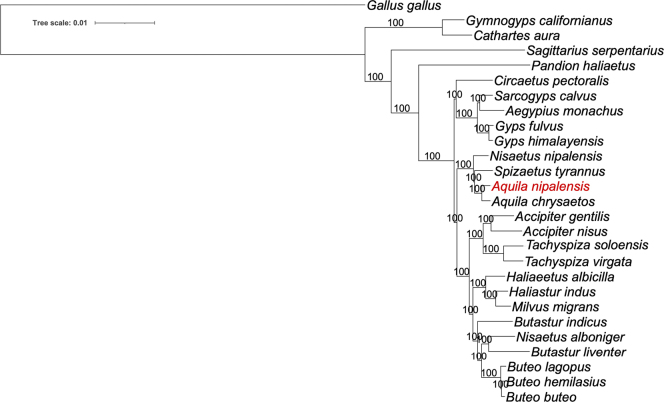
Maximum-likelihood phylogram inferred from ultraconserved elements (UCEs), based on a 100% complete data matrix across 27 avian species. *Gallus gallus* was designated as the outgroup. *Aquila nipalensis* is highlighted in red. Bootstrap support values are shown at each node.

After filtering the complete matrices at 75%, 95%, and 100% occupancy thresholds, 4,883, 4,698, and 4,535 UCE loci were retained, respectively. The resulting phylogenetic trees from all three filtered datasets exhibited identical topologies and consistent bootstrap support values, indicating that varying levels of missing data had no detectable impact on tree structure or nodal support. The analysis recovered *A. nipalensis* as sister to *A. chrysaetos*, identifying the latter as its closest extant relative within the dataset. At the genus level, *Aquila* formed a well-supported clade closely related to *Spizaetus*. These taxa were nested within a broader monophyletic clade of the family Accipitridae, which included representatives of the genera *Accipiter* Brisson, 1760; *Aegypius* Savigny, 1809; *Astur* Lacepède, 1801; *Butastur* Hodgson, 1843; *Buteo* Lacépède, 1799; *Circaetus* Vieillot, 1816; *Gyps* Savigny, 1809; *Haliaeetus* Savigny, 1809; *Haliastur* Selby, 1840; *Milvus* Lacépède, 1799; *Nisaetus* Hodgson, 1836; *Sarcogyps* Lesson, 1842; *Spizaetus* Vieillot, 1816; and *Tachyspiza* Kaup, 1844 (Fig. 1).

### Mitogenome assemblies and comparative mitogenome analysis

**Figure 2.**
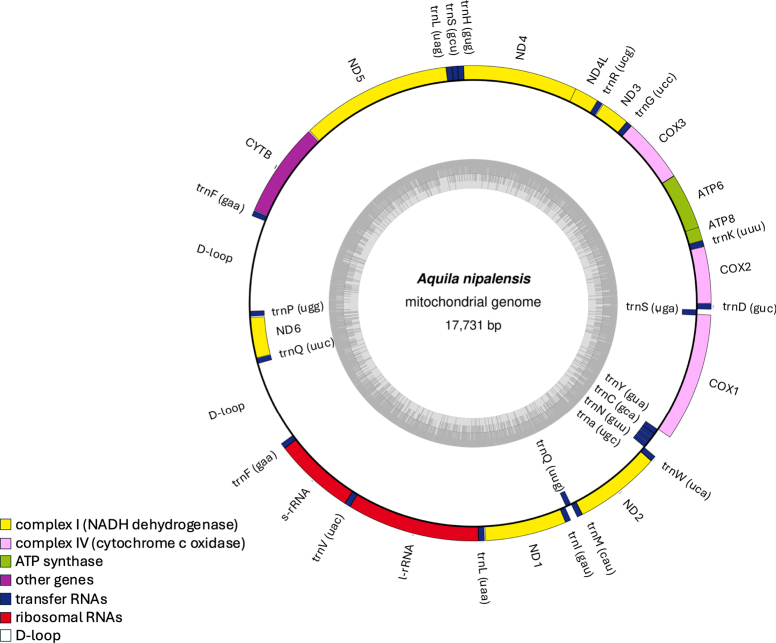
Circular map of the mitogenome of the Steppe Eagle (*Aquila nipalensis*). Annotations include genes encoding complex I (NADH dehydrogenase), complex IV (cytochrome c oxidase), ATP synthase, ribosomal RNAs, transfer RNAs, cytochrome b, and the control regions (D-loops). Genes positioned on the outer side of the circle are transcribed clockwise, whereas those on the inner side are transcribed counterclockwise. The shaded area in the inner ring represents the GC content across the mitogenome.

The mitogenome sequence has been deposited in GenBank under accession number: PV670917. The mitogenome of the Steppe Eagle was 17,731 bp in length, and the order of mitochondrial genes is illustrated in Fig. 2. The mitogenome contained 13 PCGs, 22 transfer RNA genes (tRNAs), two ribosomal RNA genes, and two control regions (D-loops). The overall nucleotide composition was 53.5% adenine and thymine (AT) and 46.5% guanine and cytosine (GC). The protein-coding regions covered 11,391 bp, accounting for 64.2% of the total mitogenome of *A. nipalensis*. All PCGs, except *ND6*, were transcribed on the plus strand. The predominant start codon was ATG, although *COX1*, *ND5*, and *ND3* utilized GTG, GTG, and ATC, respectively. Relative synonymous codon usage (RSCU) analysis revealed that the codons CCU (Pro), CCC (Pro) and UCC (Ser) were the most frequently used (Suppl. material 1: table SS1).

**Figure 3.**
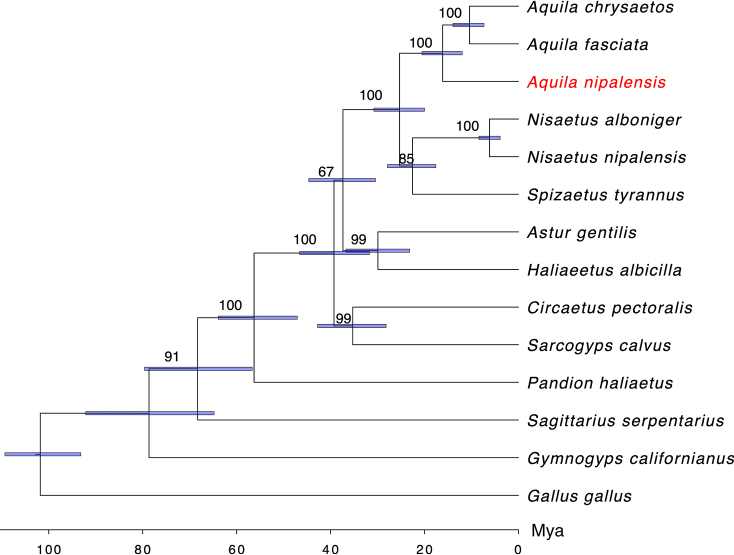
Maximum-likelihood phylogenetic analyses based on concatenated nucleotide sequences of the 13 mitochondrial protein-coding genes (PCGs) from 14 avian species. Time-calibrated phylogenetic tree with estimated divergence times (in million years ago, Mya) and bootstrap support values. *Gallus gallus* was used as the outgroup. *Aquila nipalensis* is highlighted in red.

A ML phylogenetic analysis based on concatenated nucleotide sequences of the 13 mitochondrial PCGs from 14 avian species within Accipitriformes and Cathartiformes, including *Gallus gallus* as the outgroup, is presented in Fig. 3. The corresponding phylogram with branch lengths proportional to genetic distance is shown in Suppl. material 1: fig. S1. *Aquila nipalensis* was recovered within the subfamily Aquilinae, clustering with *Aquila chrysaetos* (Linnaeus, 1758), *Aquila fasciata* Vieillot, 1822, *Nisaetus alboniger* (Blyth, 1845), *Nisaetus nipalensis* Hodgson, 1833, and *Spizaetus tyrannus* (Wied-Neuwied, 1820). The Aquilinae formed a sister clade to Accipitrinae (represented by *Astur gentilis*) and Buteonini (represented by *H. albicilla*). Divergence time estimation indicated that the most recent common ancestor of *A. nipalensis* and its closest relatives dated to approximately 15.1 Mya (95% highest posterior density [HPD]: 11.16–19.18 Mya). Fossil calibration constraints were applied to the split between *A. chrysaetos* and *G. gallus*, *Sagittarius serpentarius* and *G. californianus*, *A. chrysaetos* and *P. haliaetus*, and *A. chrysaetos* and *Astur gentilis*. Estimated divergence times across the analysed lineages ranged from approximately 5 to 90 Mya (Fig. 3). Genetic distance analysis revealed that pairwise distances between *A. nipalensis* and other *Aquila* species ranged from approximately 6.9% to 7.0%, while distances between *A. nipalensis* and species from other genera ranged from 9.7% to 27.1% (Suppl. material 1: table SS2).

### Positively selected genes

Sixteen significant PSGs were identified in *A. nipalensis*. Functional annotation indicated that these PSGs are associated with exocytosis (*EXOC7*, *SYCN*), cytoplasmic vesicle transport (*MYOF*, *SYCN*, *TGFA*), centriolar satellites (*EXOC7*, *MYOF*), and phospholipid binding (*SGIP1*, *MYOF*). The complete list of PSGs and their detailed functional annotations are provided in Tables 5, 6.

**Table 5.** Candidate positively selected genes identified in *Aquila nipalensis*.

Gene symbol	Gene name	dN/dS	P-value	FDR (*Q*-value)	Species included
SYCN	syncollin	30.23	3.93E-03	0.036	*Aquila chrysaetos*
					*Aquila nipalensis*
					*Astur gentilis*
					*Sarcogyps calvus*
TGFA	transforming growth factor alpha	20.38	1.22E-03	0.015	*Aquila chrysaetos*
					*Aquila nipalensis*
					*Astur gentilis*
					*Gallus gallus*
LOC115340598	poly(rC)-binding protein 3-like	9.76	2.41E-10	3.38E-08	*Aquila chrysaetos*
					*Aquila nipalensis*
					*Astur gentilis*
					*Gallus gallus*
LOC115352069	heat shock transcription factor, Y-linked-like	9.73	9.85E-07	4.55E-05	*Aquila chrysaetos*
					*Aquila nipalensis*
					*Astur gentilis*
					*Sarcogyps calvus*
SEBOX	SEBOX homeobox	6.80	1.10E-04	0.0020976	*Aquila chrysaetos*
					*Aquila nipalensis*
					*Astur gentilis*
					*Sarcogyps calvus*
EXOC7	exocyst complex component 7	5.69	7.52E-06	0.00024125	*Aquila chrysaetos*
					*Aquila nipalensis*
					*Astur gentilis*
					*Gallus gallus*
NEURL4	neuralized E3 ubiquitin protein ligase 4	3.87	8.96E-06	0.00024125	*Aquila chrysaetos*
					*Aquila nipalensis*
					*Astur gentilis*
					*Sarcogyps calvus*
CFAP92	cilia and flagella associated protein 92 (putative)	3.73	3.76E-07	2.02E-05	*Aquila chrysaetos*
					*Aquila nipalensis*
					*Astur gentilis*
					*Sarcogyps calvus*
PROB1	proline rich basic protein 1	3.63	2.49E-10	3.38E-08	*Aquila chrysaetos*
					*Aquila nipalensis*
					*Astur gentilis*
					*Sarcogyps calvus*
MTDH	metadherin	3.13	5.68E-03	0.0470669	*Aquila chrysaetos*
					*Aquila nipalensis*
					*Astur gentilis*
					*Gallus gallus*
LOC115351132	uncharacterized LOC115351132	2.86	2.85E-03	0.02814015	*Aquila chrysaetos*
					*Aquila nipalensis*
					*Astur gentilis*
					*Sarcogyps calvus*
SGIP1	SH3GL interacting endocytic adaptor 1	2.86	2.78E-03	0.02814015	*Aquila chrysaetos*
					*Aquila nipalensis*
					*Astur gentilis*
					*Gallus gallus*
GPAT2	glycerol-3-phosphate acyltransferase 2, mitochondria	2.64	3.82E-03	0.03623137	*Aquila chrysaetos*
					*Aquila nipalensis*
					*Astur gentilis*
					*Sarcogyps calvus*
MYOF	myoferlin	1.77	3.14E-10	3.38E-08	*Aquila chrysaetos*
					*Aquila nipalensis*
					*Astur gentilis*
					*Gallus gallus*
C1H4orf50	chromosome 1 C4orf50 homolog	1.65	5.47E-03	0.0470669	*Aquila chrysaetos*
					*Aquila nipalensis*
					*Astur gentilis*
					*Sarcogyps calvus*
DNAAF9	dynein axonemal assembly factor 9	1.37	3.90E-04	0.00617391	*Aquila chrysaetos*
					*Aquila nipalensis*
					*Astur gentilis*
					*Gallus gallus*

**Table 6.** Gene ontology enrichment analysis of candidate positively selected genes.

Category	GO term	P-value	Fold enrichment	FDR (*Q*-value)	Gene involved
UP_KW_BIOLOGICAL_PROCESS	exocytosis	0.0294	56.06	0.353	*EXOC7*, *SYCN*
GOTERM_BP_ DIRECT	exocytosis	0.0548	31.65	1.00e+0	*EXOC7*, *SYCN*
GOTERM_CC_ DIRECT	cytoplasmic vesicle	0.0567	6.81	1.00e+0	*MYOF*, *SYCN*, *TGFA*
GOTERM_CC_ DIRECT	centriolar satellite	0.0622	28.12	1.00e+0	*EXOC7*, *MYOF*
GOTERM_MF_ DIRECT	phospholipid binding	0.0631	27.73	1.00e+0	*SGIP1*, *MYOF*

## Discussion

The genome size of the Steppe Eagle, *A. nipalensis*, was estimated at 1.21 Gb, which falls within the known range for Accipitriformes (1.13–1.29 Gb) (Zhang et al. 2014a; Mead et al. 2021) and aligns with recent comparative avian genomic assessments (Catanach and Pirro 2023; Catanach et al. 2025), further supporting the conserved genome size architecture within Accipitriformes. Phylogenomic inference from all sets based on UCEs recovered *A. nipalensis* as sister to *A. chrysaetos*, supporting its placement within Aquilinae (Catanach et al. 2025). The mitogenome of *A. nipalensis* exhibits the typical avian mitochondrial gene composition and organization, including 13 protein-coding genes, 22 tRNAs, and two rRNA genes. Comparative analysis showed that its gene order is largely conserved among Accipitriformes mitogenomes reported previously (Zhou et al. 2019; Urantówka et al. 2021). Notably, the mitogenome of *A. nipalensis* contains two control regions, which differs from some earlier reports (Zhou et al. 2019) but is consistent with several accipitriform species (Urantówka et al. 2021; Sonongbua et al. 2024). Duplication of mitochondrial control regions has been associated with increased longevity in birds (Skujina et al. 2016), suggesting potential functional or life-history implications. However, further comparative analyses are required to clarify its adaptive significance in raptors.

UCE-based phylogenomic analysis provided the most robust estimate of species relationships. UCEs, distributed across the nuclear genome and biparentally inherited, capture genome-wide evolutionary history and avoid the maternal bias inherent to mitochondrial markers (Faircloth et al. 2012; McCormack et al. 2013). Across all occupancy thresholds, tree topology and bootstrap support remained consistent, indicating strong phylogenomic signal and minimal impact of missing data. Nevertheless, concatenated phylogenomic analyses may obscure gene tree heterogeneity arising from incomplete lineage sorting or hybridization, potentially leading to inaccurate species tree inference (Kubatko and Degnan 2007).

The mitogenome of *A. nipalensis* exhibits the typical avian mitochondrial gene composition and organization, including 13 protein-coding genes, 22 tRNAs, and two rRNA genes. Comparative analysis showed that its gene order is largely conserved among Accipitriformes mitogenomes reported previously (Zhou et al. 2019; Urantówka et al. 2021). Notably, the mitogenome of *A. nipalensis* contains two control regions, which differs from some earlier reports (Zhou et al. 2019) but is consistent with several accipitriform species (Urantówka et al. 2021; Sonongbua et al. 2024). Duplication of mitochondrial control regions has been associated with increased longevity in birds (Skujina et al. 2016), suggesting potential functional or life-history implications. However, further comparative analyses are required to clarify its adaptive significance in raptors. Although complete mitogenomes provided strong phylogenetic signal due to their high copy number and extensive comparative databases (Galtier et al. 2009), mitochondrial DNA represents a single maternally inherited locus and may therefore produce phylogenetic patterns that differ from nuclear genome-based hypotheses (Toews and Brelsford 2012; Mindell et al. 2018). Such discordance may arise from mitochondrial introgression, substitution saturation, or lineage-specific rate heterogeneity. Divergence time estimates differed between the 13-PCG dataset (~15.1 Mya) and the *CYTB*+*ND2* dataset (~6.9 Mya), underscoring the impact of marker choice on molecular dating. The longer concatenated 13-PCG dataset likely provided increased phylogenetic signal and reduced stochastic variance, resulting in more stable rate estimates. In contrast, shorter loci such as *CYTB* and *ND2* are more susceptible to rate heterogeneity, selective pressures, and saturation, potentially leading to biased divergence estimates (Campillo et al. 2019; Jiang et al. 2025). Despite these discrepancies in branch lengths and node ages, overall topology remained congruent across datasets, reinforcing the stability of inferred relationships within Aquilinae while emphasising caution in interpreting divergence times.

Genes under positive selection further provided insight into potential adaptive processes. *EXOC7* and *SYCN* are involved in vesicle trafficking and regulated exocytosis, processes essential for membrane targeting and cellular homeostasis (Wäsle et al. 2005; He et al. 2007). *SYCN* has also been proposed to possess antimicrobial properties (Waters et al. 2021), which may be associated with dietary exposure to pathogen-rich environments. *MYOF*, which is associated with endothelial growth and tissue development (Bernatchez et al. 2009), may contribute to cardiovascular and muscular adaptations relevant to sustained flight performance. The relatively small number of PSGs detected is likely attributable to the short divergence times among closely related accipitrid species. Broader taxonomic sampling and complementary population-genomic approaches may further enhance detection of lineage-specific adaptive signals (Nielsen et al. 2005).

## Conclusions

This study provides the high-quality *de novo* genome and complete mitogenome assembly of *A. nipalensis*, establishing an essential genomic reference for this endangered raptor. Integrating nuclear UCE-based phylogenomics with complete and partial mitochondrial datasets clarified its evolutionary position within Accipitridae and demonstrated how marker selection can influence divergence time estimation. In addition, the identification of PSGs associated with vesicle trafficking, exocytosis, and tissue development offers preliminary insights into potential adaptive mechanisms underlying physiological performance and environmental resilience. Together, these genomic resources provide a comprehensive framework for future investigations of adaptive evolution, population structure, and evidence-based conservation strategies for the Steppe Eagle.

## Additional information

### Artificial Intelligence (AI) use

The authors accept full responsibility for the content of the manuscript, including the disclosure of any use of AI.

No AI tools were used in the preparation of this manuscript.

### Author ORCIDs

W. Buthasane https://orcid.org/0009-0007-2819-9591

T. Wongsurawat https://orcid.org/0000-0002-3659-2074

P. Jenjaroenpun https://orcid.org/0000-0002-1555-401X

S. Tangphatsornruang https://orcid.org/0000-0003-2673-0012

W. Pootakham https://orcid.org/0000-0001-6721-6453

C. Sonthirod https://orcid.org/0009-0009-3116-0078

W. Kongkachana https://orcid.org/0000-0002-2356-9246

R. Sitdhibutr https://orcid.org/0000-0003-0501-2839

C. Kasorndorkbua https://orcid.org/0000-0002-2138-9954

G. Suriyaphol https://orcid.org/0000-0002-8961-5970

### Data availability

All of the data that support the findings of this study are available in the main text or Supplementary Information.

## Supplementary materials

Supplementary material 1 Genetic data Data type docx Explanation note **table SS1**. Relative synonymous codon usage (RSCU) values of mitochondrial protein-coding genes in the steppe eagle (*Aquila nipalensis*). **figure S1**. Maximum likelihood phylogram with branch lengths proportional to genetic distance based on concatenated sequences of 13 mitochondrial PCGs from 14 avian species. **table SS2**. Pairwise genetic distances between the steppe eagle (*Aquila nipalensis*) and 21 other bird species, calculated based on concatenated mitochondrial protein-coding gene sequences. https://binary.pensoft.net/file/1666817This dataset is made available under the Open Database License (http://opendatacommons.org/licenses/odbl/1.0/). The Open Database License (ODbL) is a license agreement intended to allow users to freely share, modify, and use this Dataset while maintaining this same freedom for others, provided that the original source and author(s) are credited. Wannapol Buthasane, Thidathip Wongsurawat, Piroon Jenjaroenpun, Sithichoke Tangphatsornruang, Wirulda Pootakham, Chutima Sonthirod, Wasitthee Kongkachana, Alisa Wilantho, Ratiwan Sitdhibutr, Chaiyan Kasorndorkbua, Gunnaporn Suriyaphol
