# Supplementary material for: Endangered Steppe Eagle (Aquila nipalensis) (Aves, Accipitriformes, Accipitridae) genome and mitogenome assembly: A resource for molecular evolution and comparative genomics
Source: Zookeys. 2026 Jun 3;1281:105–22. doi: 10.3897/zookeys.1281.158566 (PMC13254548; doi:10.3897/zookeys.1281.158566)
Supplement: Supplementary material 1 — Genetic data [file zookeys-1281-105_article-158566__-s001.docx]

**Supplementary material 1**

**Table S1.** Relative synonymous codon usage (RSCU) values of mitochondrial protein-coding genes in the steppe eagle (Aquila nipalensis).

| **Codon** | **Count** | **RSCU** | **Codon** | | **Count** | **RSCU** | **Codon** | **Count** | | **RSCU** | | **Codon** | | **Count** | | **RSCU** |
| --- | --- | --- | --- | --- | --- | --- | --- | --- | --- | --- | --- | --- | --- | --- | --- | --- |
| UUU (F) | 31 | 0.52 | UCU (S) | | 87 | 1.2 | UAU (Y) | 63 | | 0.69 | | UGU (C) | | 15 | | 0.6 |
| UUC (F) | 89 | 1.48 | UCC (S) | | 139 | 1.92 | UAC (Y) | 119 | | 1.31 | | UGC (C) | | 35 | | 1.4 |
| UUA (L) | 41 | 0.63 | UCA (S) | | 92 | 1.27 | UAA (*) | 106 | | 1.95 | | UGA (W) | | 55 | | 1.45 |
| UUG (L) | 38 | 0.58 | UCG (S) | | 36 | 0.5 | UAG (*) | 68 | | 1.25 | | UGG (W) | | 21 | | 0.55 |
| CUU (L) | 71 | 1.09 | CCU (P) | | 156 | 1.41 | CAU (H) | 105 | | 0.96 | | CGU (R) | | 10 | | 0.51 |
| CUC (L) | 107 | 1.64 | CCC (P) | | 145 | 1.31 | CAC (H) | 113 | | 1.04 | | CGC (R) | | 27 | | 1.37 |
| CUA (L) | 96 | 1.47 | CCA (P) | | 109 | 0.98 | CAA (Q) | 119 | | 1.46 | | CGA (R) | | 23 | | 1.16 |
| CUG (L) | 38 | 0.58 | CCG (P) | | 33 | 0.3 | CAG (Q) | 44 | | 0.54 | | CGG (R) | | 19 | | 0.96 |
| AUU (I) | 52 | 0.71 | ACU (T) | | 97 | 1.32 | AAU (N) | 65 | | 0.72 | | AGU (S) | | 22 | | 0.3 |
| AUC (I) | 95 | 1.29 | ACC (T) | | 94 | 1.28 | AAC (N) | 116 | | 1.28 | | AGC (S) | | 58 | | 0.8 |
| AUA (M) | 67 | 1.41 | ACA (T) | | 85 | 1.16 | AAA (K) | 74 | | 1.45 | | AGA (*) | | 17 | | 0.31 |
| AUG (M) | 28 | 0.59 | ACG (T) | | 17 | 0.23 | AAG (K) | 28 | | 0.55 | | AGG (*) | | 26 | | 0.48 |
| GUU (V) | 31 | 1 | GCU (A) | | 34 | 0.71 | GAU (D) | 33 | | 0.86 | | GGU (G) | | 14 | | 0.47 |
| GUC (V) | 38 | 1.23 | GCC (A) | 91 | | 1.91 | GAC (D) | | 44 | | 1.14 | GGC (G) | 37 | | 1.23 | |
| GUA (V) | 36 | 1.16 | GCA (A) | 49 | | 1.03 | GAA (E) | | 56 | | 1.3 | GGA (G) | 38 | | 1.27 | |
| GUG (V) | 19 | 0.61 | GCG (A) | 17 | | 0.36 | GAG (E) | | 30 | | 0.7 | GGG (G) | 31 | | 1.03 | |

* indicates stop codons according to the vertebrate mitochondrial genetic code.

**Supplementary material 1**

**Figure S1.** Maximum likelihood phylogram with branch lengths proportional to genetic distance based on concatenated sequences of 13 mitochondrial PCGs from 14 avian species


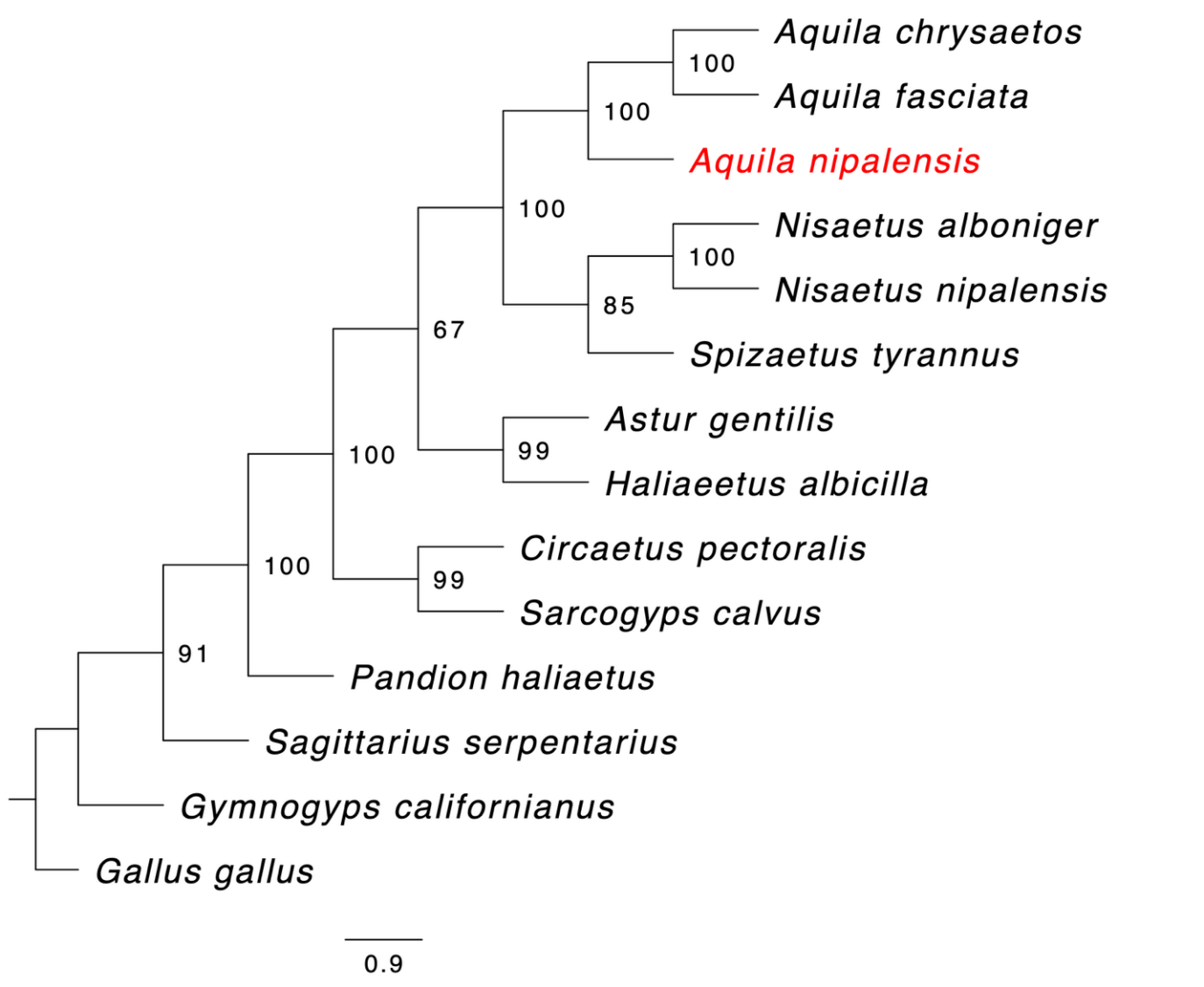


**Supplementary material 1**

**Table S2.** Pairwise genetic distances between the steppe eagle (Aquila nipalensis) and 21 other bird species, calculated based on concatenated mitochondrial protein-coding gene sequences. Distances are expressed as proportional sequence divergence (p-distance).

| **Species 1** | **Species 2** | **Genetic distance (p-distance)** |
| --- | --- | --- |
| *Aquila nipalensis* | *Astur gentilis* | 0.15224 |
| *Aquila nipalensis* | *Apaloderma vittatum* | 0.25566 |
| *Aquila nipalensis* | *Aquila chrysaetos* | 0.06879 |
| *Aquila nipalensis* | *Aquila fasciata* | 0.06979 |
| *Aquila nipalensis* | *Aquila heliaca* | 0.07014 |
| *Aquila nipalensis* | *Buceros rhinoceros silvestris* | 0.22995 |
| *Aquila nipalensis* | *Circaetus pectoralis* | 0.13652 |
| *Aquila nipalensis* | *Dryobates pubescens* | 0.25108 |
| *Aquila nipalensis* | *Falco cherrug* | 0.24154 |
| *Aquila nipalensis* | *Gallus gallus* | 0.25129 |
| *Aquila nipalensis* | *Gymnogyps californianus* | 0.19358 |
| *Aquila nipalensis* | *Halcyon senegalensis* | 0.20929 |
| *Aquila nipalensis* | *Haliaeetus leucocephalus* | 0.13838 |
| *Aquila nipalensis* | *Nisaetus alboniger* | 0.09871 |
| *Aquila nipalensis* | *Nisaetus nipalensis* | 0.09753 |
| *Aquila nipalensis* | *Pandion haliaetus* | 0.20047 |
| *Aquila nipalensis* | *Sagittarius serpentarius* | 0.20175 |
| *Aquila nipalensis* | *Sarcogyps calvus* | 0.13989 |
| *Aquila nipalensis* | *Serinus canaria* | 0.26196 |
| *Aquila nipalensis* | *Spizaetus tyrannus* | 0.10571 |
| *Aquila nipalensis* | *Tyto alba* | 0.27153 |
